# Supplementary material for: Modeling glioblastoma heterogeneity as a dynamic network of cell states
Source: Mol Syst Biol. 2021 Sep 16;17(9):e10105. doi: 10.15252/msb.202010105 (PMC8444284; doi:10.15252/msb.202010105)
Supplement: Supplementary file 5 — Source Data for Figure 3 [file MSB-17-e10105-s001.zip › Figure3A_sourcedata/GSEA_3065/hallmarks_state6.GseaPreranked.1623416308702/HALLMARK_ESTROGEN_RESPONSE_LATE.html]

Details for gene set HALLMARK\_ESTROGEN\_RESPONSE\_LATE[GSEA]

|  || Dataset | state6 |
| Phenotype | NoPhenotypeAvailable |
| Upregulated in class | na\_neg |
| GeneSet | HALLMARK\_ESTROGEN\_RESPONSE\_LATE |
| Enrichment Score (ES) | -0.33463347 |
| Normalized Enrichment Score (NES) | -1.3180617 |
| Nominal p-value | 0.06612903 |
| FDR q-value | 0.12907436 |
| FWER p-Value | 0.876 |
Table: GSEA Results Summary

  

Fig 1: Enrichment plot: HALLMARK\_ESTROGEN\_RESPONSE\_LATE      
 Profile of the Running ES Score & Positions of GeneSet Members on the Rank Ordered List

  

| PROBE | GENE SYMBOL | GENE\_TITLE | RANK IN GENE LIST | RANK METRIC SCORE | RUNNING ES | CORE ENRICHMENT || 1 | RAB31 |  |  | 23 | 0.284 | 0.0353 | No |
| 2 | PCP4 |  |  | 168 | 0.185 | 0.0435 | No |
| 3 | COX6C |  |  | 340 | 0.145 | 0.0432 | No |
| 4 | PPIF |  |  | 465 | 0.127 | 0.0459 | No |
| 5 | IMPA2 |  |  | 476 | 0.126 | 0.0616 | No |
| 6 | BAG1 |  |  | 557 | 0.117 | 0.0681 | No |
| 7 | SLC1A4 |  |  | 741 | 0.103 | 0.0607 | No |
| 8 | TOB1 |  |  | 746 | 0.102 | 0.0739 | No |
| 9 | SNX10 |  |  | 922 | 0.090 | 0.0658 | No |
| 10 | BLVRB |  |  | 928 | 0.090 | 0.0772 | No |
| 11 | HSPA4L |  |  | 1005 | 0.085 | 0.0799 | No |
| 12 | ID2 |  |  | 1058 | 0.082 | 0.0849 | No |
| 13 | RPS6KA2 |  |  | 1117 | 0.080 | 0.0889 | No |
| 14 | ALDH3A2 |  |  | 1118 | 0.080 | 0.0996 | No |
| 15 | PDCD4 |  |  | 1248 | 0.074 | 0.0946 | No |
| 16 | CCND1 |  |  | 1317 | 0.071 | 0.0962 | No |
| 17 | CKB |  |  | 1359 | 0.069 | 0.1007 | No |
| 18 | JAK1 |  |  | 1393 | 0.068 | 0.1059 | No |
| 19 | FAM102A |  |  | 1404 | 0.067 | 0.1138 | No |
| 20 | NAB2 |  |  | 1413 | 0.067 | 0.1218 | No |
| 21 | ETFB |  |  | 1467 | 0.066 | 0.1245 | No |
| 22 | PRKAR2B |  |  | 1482 | 0.065 | 0.1316 | No |
| 23 | TFAP2C |  |  | 1552 | 0.062 | 0.1319 | No |
| 24 | ITPK1 |  |  | 1637 | 0.059 | 0.1301 | No |
| 25 | RABEP1 |  |  | 1714 | 0.056 | 0.1289 | No |
| 26 | NBL1 |  |  | 1808 | 0.053 | 0.1253 | No |
| 27 | MEST |  |  | 1882 | 0.051 | 0.1238 | No |
| 28 | BTG3 |  |  | 2012 | 0.047 | 0.1153 | No |
| 29 | ARL3 |  |  | 2076 | 0.046 | 0.1141 | No |
| 30 | UGDH |  |  | 2261 | 0.040 | 0.0983 | No |
| 31 | DCXR |  |  | 2396 | 0.036 | 0.0877 | No |
| 32 | NCOR2 |  |  | 2497 | 0.033 | 0.0806 | No |
| 33 | CELSR2 |  |  | 2741 | 0.028 | 0.0564 | No |
| 34 | GALE |  |  | 2916 | 0.024 | 0.0396 | No |
| 35 | SCARB1 |  |  | 2923 | 0.024 | 0.0421 | No |
| 36 | ISG20 |  |  | 3075 | 0.020 | 0.0274 | No |
| 37 | DYNLT3 |  |  | 3160 | 0.019 | 0.0203 | No |
| 38 | MAPT |  |  | 3232 | 0.017 | 0.0144 | No |
| 39 | RBBP8 |  |  | 3390 | 0.013 | -0.0019 | No |
| 40 | PDLIM3 |  |  | 3503 | 0.011 | -0.0133 | No |
| 41 | TSPAN13 |  |  | 3733 | 0.006 | -0.0388 | No |
| 42 | MOCS2 |  |  | 3783 | 0.005 | -0.0438 | No |
| 43 | FKBP5 |  |  | 3790 | 0.005 | -0.0439 | No |
| 44 | CPE |  |  | 3921 | 0.002 | -0.0585 | No |
| 45 | PERP |  |  | 3975 | 0.001 | -0.0645 | No |
| 46 | FDFT1 |  |  | 3979 | 0.001 | -0.0647 | No |
| 47 | CDC20 |  |  | 3996 | 0.001 | -0.0664 | No |
| 48 | SLC2A8 |  |  | 4247 | -0.004 | -0.0946 | No |
| 49 | MDK |  |  | 4311 | -0.005 | -0.1012 | No |
| 50 | XBP1 |  |  | 4331 | -0.006 | -0.1026 | No |
| 51 | DLG5 |  |  | 4398 | -0.007 | -0.1093 | No |
| 52 | TIAM1 |  |  | 4472 | -0.008 | -0.1166 | No |
| 53 | FOS |  |  | 4536 | -0.010 | -0.1226 | No |
| 54 | SIAH2 |  |  | 4677 | -0.012 | -0.1371 | No |
| 55 | FLNB |  |  | 4682 | -0.012 | -0.1359 | No |
| 56 | FKBP4 |  |  | 4706 | -0.013 | -0.1368 | No |
| 57 | AFF1 |  |  | 4800 | -0.014 | -0.1456 | No |
| 58 | SLC7A5 |  |  | 4959 | -0.017 | -0.1615 | No |
| 59 | TST |  |  | 5025 | -0.019 | -0.1664 | No |
| 60 | NXT1 |  |  | 5675 | -0.032 | -0.2367 | No |
| 61 | HPRT1 |  |  | 5723 | -0.033 | -0.2377 | No |
| 62 | NRIP1 |  |  | 5868 | -0.035 | -0.2496 | No |
| 63 | CA2 |  |  | 5950 | -0.037 | -0.2540 | No |
| 64 | ABCA3 |  |  | 6089 | -0.040 | -0.2645 | No |
| 65 | HOMER2 |  |  | 6148 | -0.041 | -0.2656 | No |
| 66 | CXCL12 |  |  | 6173 | -0.042 | -0.2628 | No |
| 67 | FARP1 |  |  | 6319 | -0.045 | -0.2734 | No |
| 68 | METTL3 |  |  | 6456 | -0.049 | -0.2825 | No |
| 69 | GINS2 |  |  | 6461 | -0.049 | -0.2765 | No |
| 70 | PLXNB1 |  |  | 6476 | -0.049 | -0.2715 | No |
| 71 | CDC6 |  |  | 6482 | -0.049 | -0.2655 | No |
| 72 | ADD3 |  |  | 6818 | -0.059 | -0.2961 | No |
| 73 | OLFM1 |  |  | 6897 | -0.062 | -0.2968 | No |
| 74 | IDH2 |  |  | 6985 | -0.064 | -0.2983 | No |
| 75 | DNAJC1 |  |  | 7129 | -0.069 | -0.3055 | No |
| 76 | ELOVL5 |  |  | 7151 | -0.069 | -0.2987 | No |
| 77 | SLC26A2 |  |  | 7256 | -0.073 | -0.3009 | No |
| 78 | TOP2A |  |  | 7430 | -0.079 | -0.3103 | No |
| 79 | ABHD2 |  |  | 7643 | -0.088 | -0.3229 | Yes |
| 80 | WFS1 |  |  | 7649 | -0.088 | -0.3117 | Yes |
| 81 | DHCR7 |  |  | 7806 | -0.096 | -0.3168 | Yes |
| 82 | ATP2B4 |  |  | 7858 | -0.099 | -0.3094 | Yes |
| 83 | CA12 |  |  | 7910 | -0.102 | -0.3017 | Yes |
| 84 | AMFR |  |  | 8037 | -0.109 | -0.3016 | Yes |
| 85 | CHPT1 |  |  | 8084 | -0.113 | -0.2918 | Yes |
| 86 | CAV1 |  |  | 8156 | -0.117 | -0.2843 | Yes |
| 87 | FGFR3 |  |  | 8163 | -0.118 | -0.2692 | Yes |
| 88 | SEMA3B |  |  | 8382 | -0.144 | -0.2750 | Yes |
| 89 | GLA |  |  | 8383 | -0.144 | -0.2557 | Yes |
| 90 | IL6ST |  |  | 8394 | -0.146 | -0.2374 | Yes |
| 91 | TPBG |  |  | 8401 | -0.146 | -0.2185 | Yes |
| 92 | FABP5 |  |  | 8414 | -0.148 | -0.2001 | Yes |
| 93 | SLC16A1 |  |  | 8415 | -0.148 | -0.1803 | Yes |
| 94 | MYOF |  |  | 8488 | -0.159 | -0.1674 | Yes |
| 95 | PRSS23 |  |  | 8688 | -0.233 | -0.1591 | Yes |
| 96 | TFPI2 |  |  | 8752 | -0.290 | -0.1276 | Yes |
| 97 | CD9 |  |  | 8762 | -0.303 | -0.0882 | Yes |
| 98 | CD44 |  |  | 8778 | -0.337 | -0.0449 | Yes |
| 99 | EMP2 |  |  | 8781 | -0.350 | 0.0017 | Yes |
Table: GSEA details [plain text format]

  

Fig 2: HALLMARK\_ESTROGEN\_RESPONSE\_LATE: Random ES distribution      
 Gene set null distribution of ES for **HALLMARK\_ESTROGEN\_RESPONSE\_LATE**
